# Breakdown of Langmuir Adsorption Isotherm in Small Closed Systems

**DOI:** 10.1021/acs.langmuir.3c03894

**Published:** 2024-02-05

**Authors:** Ronen Zangi

**Affiliations:** † Donostia International Physics Center (DIPC), 20018 Donostia-San Sebastián, Spain; ‡ Department of Organic Chemistry I, University of the Basque Country UPV/EHU, 20018 Donostia-San Sebastián, Spain; § IKERBASQUE, Basque Foundation for Science, 48009 Bilbao, Spain

## Abstract

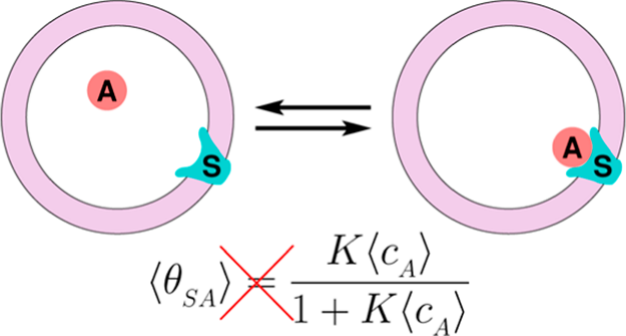

For more than a century, monolayer adsorptions in which adsorbate molecules and adsorbing sites behave ideally have been successfully described by Langmuir’s adsorption isotherm. For example, the amount of adsorbed material, as a function of concentration of the material which is not adsorbed, obeys Langmuir’s equation. In this paper, we argue that this relation is valid only for macroscopic systems. However, when particle numbers of adsorbate molecules and/or adsorbing sites are small, Langmuir’s model fails to describe the chemical equilibrium of the system. This is because the kinetics of forming, or the probability of observing, occupied sites arises from two-body interactions, and as such, ought to include cross-correlations between particle numbers of the adsorbate and adsorbing sites. The effect of these correlations, as reflected by deviations in predicting composition when correlations are ignored, increases with decreasing particle numbers and becomes substantial when only few adsorbate molecules, or adsorbing sites, are present in the system. In addition, any change that augments the fraction of occupied sites at equilibrium (e.g., smaller volume, lower temperature, or stronger adsorption energy) further increases the discrepancy between observed properties of small systems and those predicted by Langmuir’s theory. In contrast, for large systems, these cross-correlations become negligible, and therefore when expressing properties involving two-body processes, it is possible to consider independently the concentration of each component. By applying statistical mechanics concepts, we derive a general expression of the equilibrium constant for adsorption. It is also demonstrated that in ensembles in which total numbers of particles are fixed, the magnitudes of fluctuations in particle numbers alone can predict the average chemical composition of the system. Moreover, an alternative adsorption equation, predicting the average fraction of occupied sites from the value of the equilibrium constant, is proposed. All derived relations were tested against results obtained by Monte Carlo simulations.

## 1 Introduction

Adsorption, the process in which molecules A, say in a gas phase, adsorb onto sites S (here, taken with single occupancy) of a (e.g., solid) surface can be described by the following chemical equation
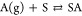
1

Assuming ideal behavior of all components, which also implies no multilayer formation, the equilibrium properties of the system, such as average fraction of occupied sites ⟨θ_SA_⟩, are well described by the celebrated Langmuir adsorption isotherm^1^
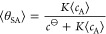
2where ⟨*c*_A_⟩ is average concentration of gas particles at equilibrium, *K*, the equilibrium (Langmuir) constant of the reaction, and *c*^⊖^, the standard (reference) concentration of adsorbate gas, introduced here to comply with the convention of rendering *K* unitless. Although the adsorption process in eq 1 is chosen to take place from a gaseous to a solid phase, a corresponding adsorption process of solutes from solution onto an interface formed at contact with solid, liquid, or gas phases yields the same Langmuir equation (eq 2). It should be pointed out that in case the adsorbate molecules are dissolved in a liquid, the change in adsorbate–solvent interactions upon adsorption is accounted for by an effective adsorption energy.^2^ This effective energy can also include possible changes in vibrational energies of the surface induced by the adsorption. A vast number of studies, encompassing different scientific fields, confirm that systems adhering to assumptions mentioned above do obey Langmuir’s equation.^3−20^ Whereas nonideal systems, for example, those characterized by substantial interactions between the adsorbed molecules and thereby represented by a modified equation of state,^21−23^ exhibit certain degree of deviations.^24^ In practice, to examine compliance with Langmuir’s isotherm, experiments with different amounts of adsorbate A are performed where its unadsorbed concentration and amount adsorbed, both at equilibrium, are measured. Then, these measured data points are fitted to the relation in eq 2, either in its nonlinear or in one of its linear forms,^25−27^ aiming to extract the value of *K*, and sometimes, the total number of adsorbing sites, N_S_^total^ = ⟨*N*_SA_⟩/⟨θ_SA_⟩. Note that none of the assumptions made in deriving Langmuir equation^1,28^ imposes conditions on the size of the system, or alternatively, on the particle numbers of the adsorbate and/or adsorbing sites. Thus, eq 2 is implied to be valid for any system size, also for those composed of only few A molecules or only few S sites.

Yet, Polak and Rubinovich argued that adsorption under nanoconfinement exhibits equilibrium properties deviating from those predicted by Langmuir’s model due to an entropic effect,^29^ and Ramaswamy et al. argued that rate equations are qualitatively incorrect in subcritical volumes.^30^ Furthermore, single-molecule experiments of small-sized systems undergoing association reactions (where both reactants are mobile in space) find that concentrations of bound complexes do not agree with predictions of the conventional chemical equilibrium theory.^31−40^ Similar behavior was also reported by computational studies.^41−56^ In light of these findings, we recently demonstrated that for bimolecular reactions, averages of quantities observed at small (finite) systems are different from those observed at large or macroscopic systems.^57−59^ This inhomogeneous character of the functions describing the system’s properties is applicable for closed systems, that is, for systems in which the total numbers of particles are fixed, such as the canonical ensemble. Then, by definition, as time or configurations are propagated, the particle numbers of all components are subjected to fluctuations with relative magnitudes that increase as system’s size decreases. In fact, from the magnitudes of these fluctuations alone, it is possible to determine average properties of the system including the number (or concentration) of bound particles.

What is the difference then between small and large systems? Because we are dealing with bimolecular reactions, which necessarily proceed via two-body interactions, cross-correlations in particle numbers (or concentrations) must be taken into account when describing mass-actions at equilibrium.^57−60^ The importance of these cross-correlations is augmented as particle numbers and/or volume decrease, as well as, for lower temperatures or larger binding energies, and the amplitude of their effect can reach few orders of magnitude. On the other hand, when the system is large enough (hereafter, will be used interchangeably with the term macroscopic), these cross-correlations are negligible and can be completely ignored. Therefore, the known thermodynamic relations in chemical equilibrium, observed to hold for macroscopic systems, are only private cases of a general formalism that permits fluctuations in the system.

**Figure 1:**
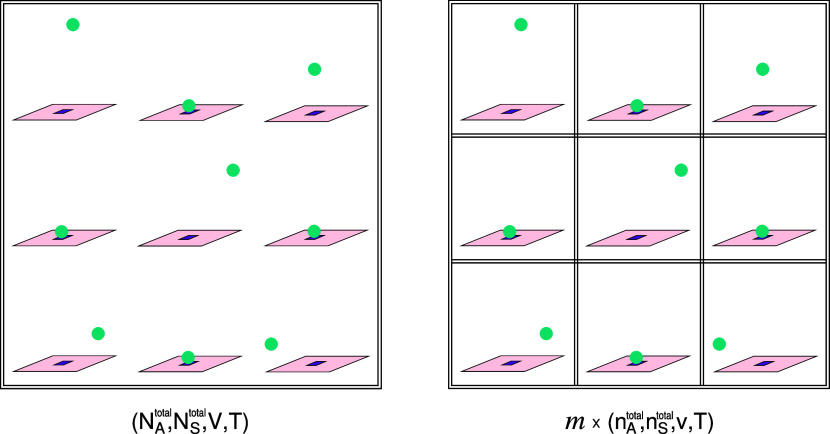
Left: a single large-sized system describing adsorption (eq 1) in the canonical ensemble (*N*_A_^total^, *N*_S_^total^, *V*, *T*). Right: *m* isolated and independent small systems representing the same process where each system is in the canonical ensemble (*n*_A_^total^, *n*_S_^total^, *v*, *T*). The A gas molecules are depicted by green balls, and the adsorbing S sites are depicted by purple squares. The fixed parameters of the systems on the left and right are related by *N*_A_^total^ = *m*·*n*_A_^total^, *N*_S_^total^ = *m*·*n*_S_^total^, and *V* = *m*·*v*.

Following the discovery of the law of mass action,^61^ Langmuir invoked kinetics arguments to derive eq 2 and expressed the rate at which the A molecules adsorb onto the surface (the forward reaction in eq 1) as , where θ_S_ is the fraction of unoccupied sites. We further emphasize that the values of *c*_A_ and θ_S_ correspond to values at equilibrium, each averaged independently either over the duration of the measurements or over the ensemble of configurations. This is because only when these quantities are considered uncorrelated, can the derivation proceed to yield eq 2. Applying our above-mentioned argument of the necessity to include cross-correlations also here, that is for expressing the bimolecular reaction rate, we claim in this paper that for small systems, eq 2 is not valid and another relation holds. Rephrased differently, consider two systems representing the adsorption process of eq 1 as sketched in Figure 1. On the left, a single large system in the canonical ensemble (*N*_A_^total^, *N*_S_^total^, *V*, *T*) is depicted, whereas on the right, *m* isolated and independent small systems are shown, each of which is described by its own canonical ensemble (*n*_A_^total^, *n*_S_^total^, *v*, *T*). It is argued here that even if *N*_A_^total^ = *m*·*n*_A_^total^, *N*_S_^total^ = *m*·*n*_S_^total^, and *V* = *m*·*v*, averages obtained in the large system on the left are not equal to those obtained by the *m* small systems on the right. Nonetheless, it is possible to transform averages observed at small systems to their corresponding values at macroscopic systems and vice versa. This can be performed by utilizing the equilibrium constant that, when accounts for cross-correlations in concentrations, has the same value independent of system’s size, a property enabling it to link the chemical compositions of the two systems at equilibrium.

We start by deriving a general expression of the equilibrium constant for adsorption.

## 2 Results and Discussion

### 2.1 Derivation of the Equilibrium Constant for Adsorption

We consider the adsorption process specified in eq 1 as associations between gas particles A and immobile (surface) particles S to produce immobile bound products SA. It is assumed that all components behave ideally, which means that, except for the adsorption reaction described in eq 1, the particles do not interact with one another and a single adsorbing site can only interact with a single gas particle. In other words, adsorption on a given site does not affect adsorptions on nearby sites and no multilayer adsorption is possible.

To obtain the expression of the equilibrium constant, *K*, at temperature *T*, we utilize the definition
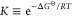
3where *R* is the gas constant and Δ*G*^⊖^, the standard Gibbs energy change of adsorption, is the change in the Gibbs free energy when 1 mol of A adsorbs onto 1 mol of S vacant sites to produce 1 mol of occupied sites, under conditions in which both reactants and product are at their standard (reference) states. For a gas component, the standard state is normally defined by a chosen value of its partial pressure, *P*^⊖^, nevertheless, we find it convenient to specify instead the corresponding standard concentration, *c*^⊖^. If *N*^⊖^ is the number of A particles which adsorb onto *S* sites when the reference reaction goes into completion (which at this point is not restricted to be 1 mol but only a large number), then the volume of the gas is *V*^⊖^ = *N*^⊖^/*c*^⊖^. The standard states of the immobile (vacant and occupied) sites are not consistently defined in the literature. This introduces no problem as long as these two standard states are the same. To advance with the derivation, we choose their standard state to correspond to the particle number *N*^⊖^. This can be expressed, for example, by surface density or concentration of the vacant/occupied sites, *N*^⊖^/*A*_S_, where *A*_S_ is the surface area of the adsorbent.

Applying a statistical mechanics framework, the reference system is chosen to be described by the canonical ensemble (*N*_A_^⊖^, *N*_S_^⊖^, *V*^⊖^, *T*) where *N*_A_^⊖^ = *N*_S_^⊖^ = *N*^⊖^ are the number of A particles and S sites. We consider *V*^⊖^ to correspond also to the volume of the whole system by assuming that the excluded volumes of the A particles and S sites are negligible. The corresponding partition function can be expressed by
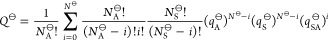
4where summation over index *i* (*i* ≡ *N*_SA_) includes all possible numbers of occupied SA sites, and thereby, all possible (interparticle) energy states. *q*_A_^⊖^ and *q*_S_^⊖^ are single-particle partition functions of an A particle in the gas phase and of a vacant S site, both, in the reference system. *q*_SA_^⊖^ is the pair-particle partition function of an occupied SA site (also in the reference system) which incorporates the Boltzmann factor of the adsorption energy. The division, outside the sum, by *N*_A_^⊖^! is because the A gas particles are indistinguishable. In contrast, the immobile adsorbing sites S are distinguishable, and therefore, a corresponding division by *N*_S_^⊖^! is not performed. The first and second fractions of factorials inside the sum express the degeneracy of state *i*. The first term counts the number of ways to choose *i* A particles out of *N*_A_^⊖^ particles where the order in the chosen group is not important. The second term represents the number of ways to distribute these *i* A particles into *N*_S_^⊖^ sites. Even though in the reference system *N*_A_^⊖^ = *N*_S_^⊖^ = *N*^⊖^, we kept indicating the subscripts of the particle numbers in the terms of the factorials in eq 4 to clarify their origin. Otherwise we obtain
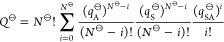
5

Equation 5 is arranged in such a way that each (single- or pair-) particle partition function, raised to the power of its particle number, is divided by the factorial of this power. Yet, it is worth emphasizing that this division does not imply that the S or SA sites are indistinguishable, but instead, it is a consequence of their equivalence (degeneracy in the energy of the state). In fact, the distinguishability of the S sites (either vacant or occupied) is manifested by the existence of the factor *N*^⊖^! outside the sum, which is absent for binding reactions where both reactants are indistinguishable.^57^

We continue by expressing the Gibbs free energy change, , when *N*^⊖^ particles of A adsorb onto *N*^⊖^ sites *S*. Then, Δ*G*^⊖^ is obtained by scaling  to 1 mol. In a canonical ensemble, the partition function of the system is related to the Helmholtz free energy. Therefore, the corresponding change in the Helmholtz free energy, , can be calculated from the ratio of the probability to find the system in the fully adsorbed state, *p*^SA^ (i.e., the fraction of the state *i* = *N*^⊖^ in the sum of the partition function in eq 5), to the probability of the fully unadsorbed (or vacant) state, *p*^A+S^ (the fraction of the state *i* = 0). Note that the reference system is implied to be macroscopic as it reports a change in the Gibbs energy per mole of stoichiometric reaction. This is the reason we restricted *N*^⊖^ to be large. Thus, we can use the thermodynamic relation between Gibbs and Helmholtz free energies and write  as
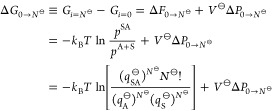
6where  is the change in the pressure of the system when *N*^⊖^ A gas particles are adsorbed. Noting  equals –*N*^⊖^*k*_B_*T* for ideal gases and applying Stirling’s approximation to evaluate ln *N*^⊖^! (again, justified because *N*^⊖^ is large), we get
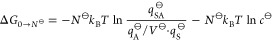
7an expression that is the same as that obtained for binding reactions when both reactants are mobile indistinguishable particles.^57^ This is because the term in eq 5 characterizing the distinguishability of the immobile *S* sites cancels-out when calculating the ratio of probabilities in eq 6. Hence, from here, the derivation of the expression of *K* is similar to that for a binding reaction; nonetheless, we will briefly outline the critical steps.

In the reference system, we looked only at two states, *i* = 0 and *i* = *N*^⊖^, from which Δ*G*^⊖^ is to be calculated. This reference reaction is hypothetical in the sense that full conversion is, in general, not attainable spontaneously. It turns out, we can evaluate Δ*G*^⊖^ of this reference system from equilibrium properties, spontaneously attainable, of a similar system at the same temperature but with arbitrary concentrations and size, which can be macroscopic or finite. The canonical ensemble of the arbitrary system is specified by the parameters (*N*_A_^total^, *N*_S_^total^, *V*, *T*), where *N*_A_^total^ = *N*_A_ + *N*_SA_ and *N*_S_^total^ = *N*_S_ + *N*_SA_ are total numbers of A particles and S sites, which are in general not equal. Its partition function is similar to eq 4 and takes the form
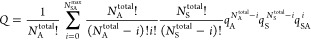
8where *N*_SA_^max^ is the maximum number of occupied sites that the system can support (i.e., *N*_SA_^max^ = *N*_A_^total^ for *N*_A_^total^ ≤ *N*_S_^total^, or *N*_SA_^max^ = *N*_S_^total^ otherwise).

To calculate Δ*G*^⊖^ by eq 7 requires the evaluation of the ratio *q*_SA_^⊖^*V*^⊖^/(*q*_A_^⊖^*q*_S_^⊖^). Being fixed in space, it is clear that *q*_S_^⊖^ and *q*_SA_^⊖^ are equal to the corresponding particle partition functions of the arbitrary system, *q*_S_ and *q*_SA_. In contrast, due to translation, the single-particle partition function of A gas particle depends on the volume of the gas. If we approximate the discrete sum of quantum translational energy states by an integral,^58^ the dependency of this single-particle partition function (both of the reference and the arbitrary systems) on volume can be shown to be linear and the following equality exists
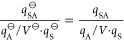
9

The validity of approximating the discrete sum with an integral is decreased with decreasing temperature, mass, and volume. However, it is shown to be well justified for almost all molecular systems at relevant conditions.^58^ We proceed by multiplying and dividing the ratio on the right-hand side of eq 9 by the term
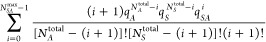
10and obtain
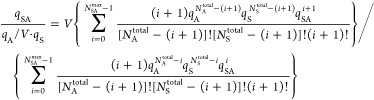
11

By applying a sequence of algebraic operations on the right-hand side of eq 11 (without introducing any further assumptions), it can be shown that^57^
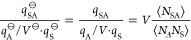
12the ratio of particle partition functions reduces to a ratio of average number of occupied sites to the average of product between the number of unadsorbed A gas and number of vacant S sites, where both averages are taken at equilibrium conditions of the arbitrary system. Inserting the equality of eq 12 into eq 7 and scaling  to 1 mol yield
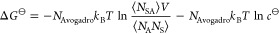
13from which *K* is obtained using its definition in eq 3
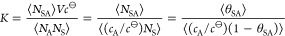
14where θ_SA_ = *N*_SA_/*N*_S_^total^ is the fraction of occupied sites. The expression of *K* in eq 14 is different from that derived in textbooks and routinely utilized in the literature. The difference is that the latter ignores correlations between the reactant’s particle numbers/concentrations and is written as^62,63^
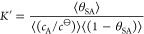
15

This neglect of cross-correlations is significant for small systems and renders the equilibrium constant *K* not constant for systems at the same temperature but with different concentrations or sizes. The discrepancy of *K*′ from *K* can reach few orders of magnitude and is augmented for lower temperatures or stronger binding/adsorption energy, as well as for higher concentrations. With increasing system size, *K*′ approaches *K*, and for macroscopic systems, these correlations in reactant’s concentrations can be ignored.

### 2.2 Validations against Monte Carlo Simulations

We now test the predictions derived above against results obtained by Monte Carlo (MC) simulations. In short, we performed four series of simulations. In the first, R1, we increased *N*_S_^total^ = *N*_A_^total^ from 1 to 120 and simultaneously increased the volume in such a way that the concentration *c*_A_^total^ = *N*_A_^total^/*V* is constant at ∼0.013 M. The series R2 and R3 involved variations in the particle number of only one of the reacting species (either A gas or S adsorbing site), whereas the number of the other reactant was fixed. In these cases, the volume also changed, subjected to maintain the concentration of the most abundant species constant (∼0.025 M). More information about the systems, model particles, and computations is given in the Materials and Methods section.

**Figure 2:**
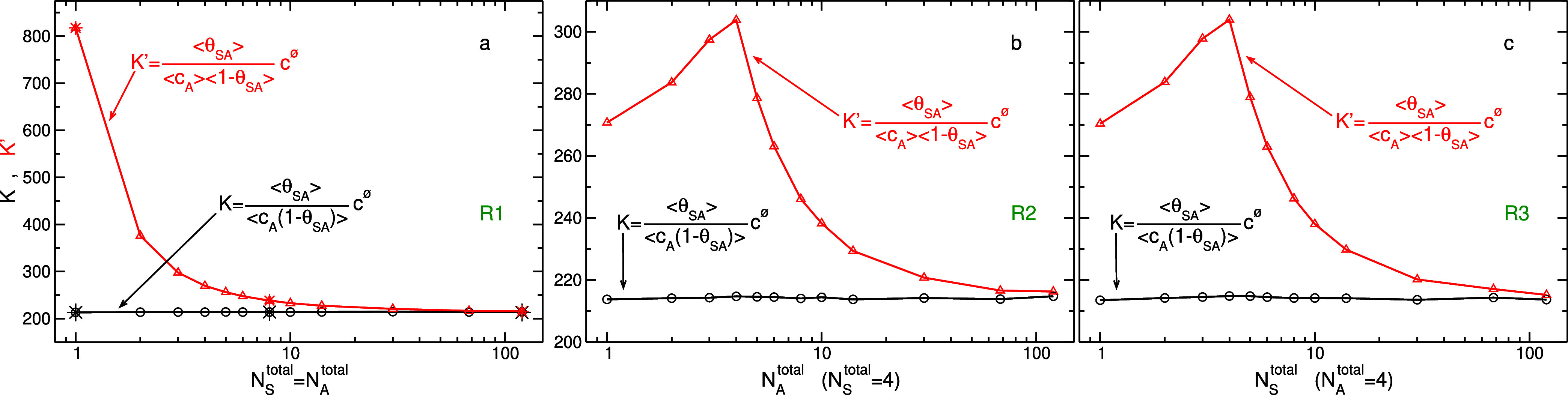
Equilibrium constant of adsorption *K*, defined in eq 14, for (a) R1 series of simulations as a function of total number of immobile (S) and mobile (A) particles, *N*_S_^total^ = *N*_A_^total^, (b) R2 series as a function of *N*_A_^total^, where *N*_S_^total^ is fixed, and (c) R3 series as a function of *N*_S_^total^, where *N*_A_^total^ is fixed. For comparison, the conventional expression of the equilibrium constant ignoring two-body correlations *K*′, defined in eq 15, is also displayed. The curves of *K*′ in (b,c) seem identical, nonetheless, they are distinct and were obtained independently. The star symbols in (a) at *N*_S_^total^ = *N*_A_^total^ = 1, 8, 120 correspond to additional simulations in which the S (along with the A) particles are mobile.

In Figure 2, we display the equilibrium constant for R1–R3 series of simulations. As should be the case, the value of *K* computed by eq 14 is constant for all systems of the three series. Due to different scales of the *y*-axis, it might be difficult to notice that the average of *K* for all points in R1, 214.0 ± 0.3, is very similar to those for R2 and R3, 214.3 ± 0.3 and 214.2 ± 0.4, respectively. In contrast, the value of *K*′ (eq 15) is not constant and varies significantly with system’s size and concentration of *N*_S_^total^ or *N*_A_^total^. Only at large system sizes, the value of *K*′ approaches that of *K* and apparently it happens “faster” in R1 series, compared to R2 and R3, likely because the concentration is lower. Note that the maxima observed in R2 and R3, at *N*_A_^total^ = 4 and *N*_S_^total^ = 4, are because for smaller particle numbers, the most abundant species is that whose particle number is fixed, whereas, for larger particle numbers, it is that with varying particle number. To compare the equilibrium constant of adsorption, where one reactant is mobile and the other is immobile, to that of binding, where both reactants are mobile, we repeated three points in R1 series, *N*_S_^total^ = *N*_A_^total^ = 1, 8, 120, but allowed the S particles to freely move in the simulation box. The results, displayed in Figure 2 by star symbols, indicate that the values of *K* (as well as *K*′) are almost identical to those obtained by simulations of the adsorption process. Again, this is because *K* is described by the ratio of probabilities of observing two states, and the reduced phase space (or distinguishability) in the system cancels-out when taking this ratio.

Given two variables of a system, ζ and η, it is well known from statistical mechanics that the average amplitude of their cross fluctuations relative to their mean values, *l*(ζ,η) = ⟨(ζ – ⟨ζ⟩)(η – ⟨η⟩)⟩/(⟨ζ⟩⟨η⟩), decreases linearly with system’s size.^64^ Furthermore, these average fluctuations can be related to some properties, such as heat capacity, of the system.^65,66^ In relation to bimolecular association reactions, it was shown that the average number of bound product is inversely proportional to two relative fluctuations in the system,^57^ which can be projected on the adsorption reaction described in eq 1 to yield
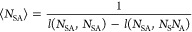
16

**Figure 3:**
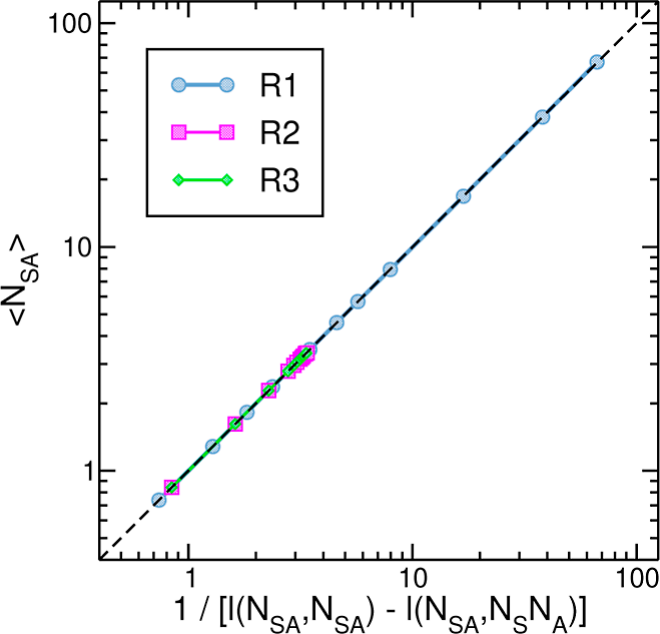
Relation between the average number of adsorbed particles and the reciprocal of a difference between two relative fluctuations (eq 16). The dashed black line corresponds to *y* = *x* and is shown as a reference for perfect agreement. The points of R2 series almost overlap those of R3.

In Figure 3, we examine this relation on R1–R3 series of simulations. The results, with points spanning approximately two orders of magnitude in values, indicate an excellent agreement with the theory.

### 2.3 Prediction of Surface Coverage from the Equilibrium Constant

Even though the equality in eq 16 provides a route to predict the average number of occupied sites from fluctuations in the system, there are benefits to establish an alternative relation in which the required quantities do not need to be extracted from the system in question. In effect, this is the reason why the equilibrium constant is so important; its value and the parameters specifying a desired system (e.g., *N*_A_^total^, *N*_S_^total^, *V*, *T*) can predict the chemical composition of that system. This is well known for macroscopic systems where the solution for θ_SA_ in eq 15 is straightforward and yields the Langmuir adsorption isotherm equation^1^ shown in eq 2.

**Figure 4:**
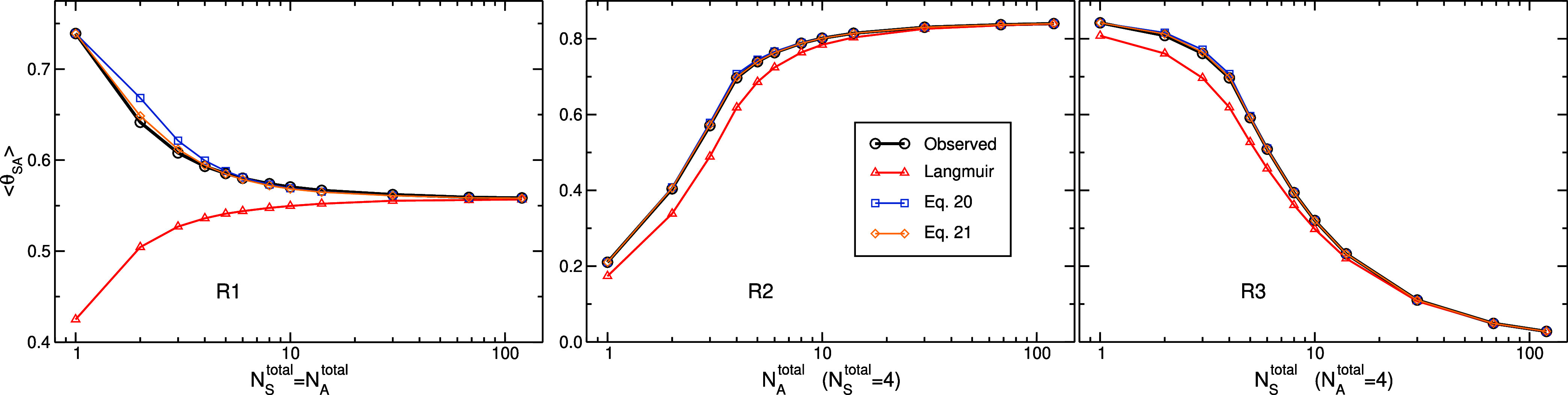
Predictions of average number of occupied adsorbing sites, presented here as average fraction ⟨θ_SA_⟩ = ⟨*N*_SA_⟩/*N*_S_^total^, from the value of *K* and parameters specifying the system. Results based on the Langmuir adsorption isotherm (red, triangles) are calculated by eq 2 where the value of *K* is that determined for a macroscopic system. Also displayed are results based on eq 17 using an approximation to evaluate *l*(*N*_SA_, *N*_SA_) (eq 19). In a previously proposed empirical relation,^57^ the value of λ appearing in eq 19 is given by eq 20 (blue, squares), whereas in current work, it is proposed to be given by eq 21 (orange, diamonds). Values of ⟨θ_SA_⟩ observed directly in the simulations are shown as references (black, circles).

In Figure 4, we display the average fraction of occupied sites, ⟨θ_SA_⟩, observed in the simulations for R1–R3 series. Predictions based on the Langmuir adsorption isotherm indicate that for R2 and R3 series, the predicting curves deviate moderately from the curve determined by direct counting from the simulation of each system. In fact, the shapes of the curves are similar and at large numbers of particles (either *N*_A_^total^ in R2 or *N*_S_^total^ in R3), the predictions are excellent. Very good predictions are also exhibited in the R1 series at the two largest numbers of particles; however, for smaller numbers, significant discrepancies are observed with magnitudes intensifying as *N*_S_^total^ = *N*_A_^total^ decreases. For example, the observed value of ⟨θ_SA_⟩ in the simulation at *N*_S_^total^ = *N*_A_^total^ = 1 is 0.74, whereas the Langmuir equation predicts a value of 0.42.

In principle, one can solve for ⟨θ_SA_⟩ in eq 14, however, because of cross-correlations in particle numbers of A and S, this is not so simple. Yet, it is easy to show that
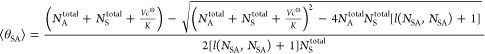
17where *l*(*N*_SA_, *N*_SA_) are relative fluctuations in the number of occupied sites. This means that the average number ⟨*N*_SA_⟩ can be calculated from its spread. For macroscopic systems, *N*_S_^total^, *N*_A_^total^ → ∞, we know *l*(*N*_SA_, *N*_SA_) → 0, and ⟨θ_SA_⟩ can be easily obtained from eq 17. The other extreme case, which is also solvable, is when the total number of, at least, one component equals one. In these systems, ⟨*N*_SA_^2^⟩ = ⟨*N*_SA_⟩, and therefore, the relative fluctuations are related to *K* by the (exact) relation
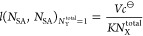
18where *X* refers to the more abundant component, *N*_X_^total^ ≥ *N*_Y_^total^, regardless being the gas particles or the immobile adsorbing sites.

Based on the behavior of *l*(*N*_SA_, *N*_SA_) described in eq 18 and in the thermodynamic limit, in a previous publication, we suggested an empirical interpolation applicable for all possible particle numbers^57^
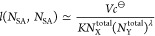
19where 
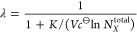
20

Thus, eqs 19 and 20 can be used together with eq 17 to yield an approximation for the average fraction of occupied sites from only the value of *K* (and the parameters specifying the system). The results, shown in Figure 4, exhibit very good agreement with values observed directly in the simulations, and for finite systems, significantly improve the predictions calculated by the Langmuir equation. Nevertheless, for some points, *N*_S_^total^ = *N*_A_^total^ = 2, 3, 4 in the R1 series, the predictions are noticeably imperfect. That being so, we re-evaluated empirically the suggested value of λ and found an alternative expression that predicts better the observed results
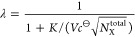
21

The results of this new approximation are shown in Figure 4 as well, demonstrating excellent agreement relative to direct counting with almost unnoticeable discrepancies. In order to test whether the new approximation to evaluate *l*(*N*_SA_, *N*_SA_) given in eq 21 would also improve the predictions made in a previous work, we applied it for binding reactions for all systems investigated previously. The results are presented in Figures SI-1 and SI-2 of the Supporting Information. In all 52 systems examined, agreement with direct counting is excellent, and in all points where previous approximation (eq 20) displayed noticeable discrepancies, predictions based on current approximation (eq 21) offer significant and satisfactory improvements.

**Figure 5:**
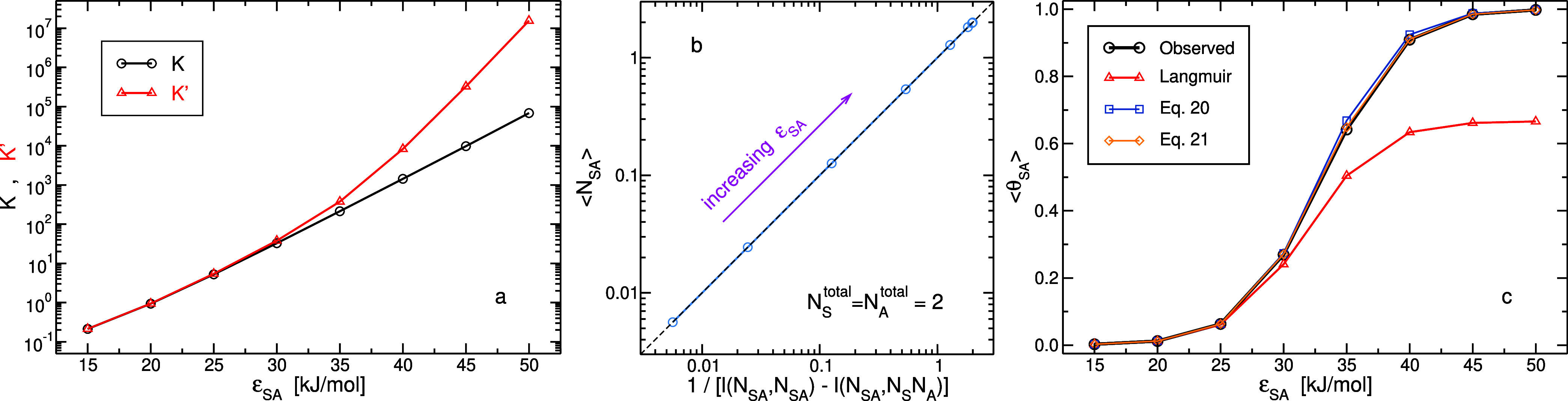
Results from R4 series of simulations in which the adsorption energy (ϵ_SA_ = 35 kJ/mol in R1–R3) is systematically modified in the range of 15–50 kJ/mol. In all R4 systems, *N*_S_^total^ = *N*_A_^total^ = 2 and *c*_A_^total^ = 0.008 molecules/nm^3^. (a) Equilibrium constant, *K* (eq 14), and the conventional expression ignoring correlations, *K*′ (eq 15), as a function of ϵ_SA_. (b) Relation between average number of occupied sites and relative fluctuations (eq 16). Note that at ⟨*N*_SA_⟩ ≃ 2, there are two points that are almost completely overlapping. (c) Predictions of average fraction of occupied sites as a function of ϵ_SA_. Colors and symbols of the different curves are the same as those in Figure 4.

The systems in R1–R3 series were all performed with the same strength of adsorption energy, which means that when combined with conditions of constant temperature, the resulting equilibrium constant is the same for all systems. Therefore, in order to test the performance of the proposed predictions for a range of values of *K*, we performed a fourth series of simulations, R4, wherein the well depth of the Lennard-Jones (LJ) potential between the gas particles and the adsorbing sites is modified systematically from 15.0 to 50.0 kJ/mol in equal steps of 5.0 kJ/mol. We chose the finite system of *N*_S_^total^ = *N*_A_^total^ = 2 because it displayed the largest discrepancies with our predictions (pointing out once again that the private case in which the particle number of, at least, one of the components equals one can be solved exactly). As shown in Figure 5a, the variations in the strength of the adsorption energy produce equilibrium constants that range from 2 × 10^–1^, for the weakest interaction, to 7 × 10^4^, for the strongest interaction.

Substantial deviations of *K*′ from *K* start at around ϵ_SA_ = 30 kJ/mol and rapidly intensify with an increase in the adsorption energy. For example, the relative deviation, (*K*′ – *K*)/*K*, is 1.4×10^–3^ for ϵ_SA_ = 15 kJ/mol, whereas, it is 230 for ϵ_SA_ = 50 kJ/mol. In Figure 5b, the relation between the average number of occupied sites and the reciprocal of a difference between two relative fluctuations in the system, as described in eq 16, is plotted. The results indicate an almost perfect agreement. Moreover, the predictions of computing ⟨θ_SA_⟩ from *K* are examined in Figure 5c. The Langmuir adsorption isotherm model predicts the occupancy very well at weak adsorption energies (or high temperatures) but fails when the adsorption is strong (low temperatures). In fact, the discrepancies of the predictions reflect the deviations of *K*′ from *K*. Predicting ⟨θ_SA_⟩ by approximating *l*(*N*_SA_, *N*_SA_) (eq 18) using λ given by eq 20 is very good even at strong adsorption energies. Nonetheless, when λ is given by eq 21, the predictions are further improved and almost coincide with direct counting.

Taken together, the results presented in Figures 2, 4, and 5 point also to the complexity of assigning a priori a minimum size to a system, above which it behaves macroscopically. The reason is that this minimum size depends on five parameters. Two of these parameters, temperature and adsorption energy, can be represented by a single parameter, the reduced temperature *k*_B_*T*/ϵ_SA_. The thermodynamic limit is hence approached by increasing this reduced temperature, number of particles *N*_S_^total^ and *N*_A_^total^, and volume *V* (see Figure 1b in a previous work^57^). The extent to which the term *K*′/*K* – 1 approaches zero can then serve as a descriptor for macroscopic behavior, and a choice of a threshold value classifies the system as macroscopic or finite. By definition, the term *K*′/*K* – 1 equals *l*(*N*_A_, *N*_S_). However, our attempts to relate these relative fluctuations to the four parameters mentioned above met with no success.

On a last note, the curve of ln *K* as a function of ϵ_SA_ shown in Figure 5a is almost linear (linear regression yields correlation coefficient of 0.9993). This is because the two-body particle partition function of an occupied site, *q*_SA_, contains the factor , where *U*_SA_ is the effective adsorption energy, proportional to ϵ_SA_ but with a negative sign. In case −*U*_SA_ = ϵ_SA_, the slope of the line equals 1/*RT* = 0.401 mol/kJ. However, the linear regression of the simulation data points yields a slope of 0.366 mol/kJ. We conjecture that this difference, as well as the small deviation of the correlation coefficient from 1, arises due to changes in vibrational energy of an occupied site with changes of ϵ_SA_.

We now discuss four points related to the theoretical derivation of adsorption equilibrium in small systems.

### 2.4 Discussion

#### 2.4.1 Chemical Equilibrium of Adsorption in Open Systems

Derivation of Langmuir adsorption isotherm, within a statistical mechanics framework, is customarily performed in the literature by the grand-canonical ensemble.^28^ In this ensemble, the chemical potential of the adsorbate, μ_A_, is constant by coupling the system to a bulk reservoir of A, whereas its particle number (or concentration) is subjected to fluctuations. Yet, for ideal systems, the familiar relation between chemical potential, relative to that at the standard state, and concentration
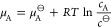
22implies that fixing the chemical potential necessarily fixes the concentration. This situation obviously holds in the thermodynamic limit, because if *N*_A_ is large enough and the system is completely open to a bulk reservoir of A, the variations in concentration (due to adsorptions and desorptions at equilibrium) are very small and can be rapidly compensated by diffusion of A between the reservoir and system. Then, the properties of this macroscopic system described by the grand-canonical ensemble are the same as those obtained by the canonical ensemble because the cross-correlations in particle numbers depicted in eq 14 are decoupled, yielding Langmuir’s equation(eq 2).

Consider now a small open system where the diffusion relaxation time of the adsorbate A between the small system and the bulk reservoir is slower than adsorption/desorption times. This situation can happen if the system is defined as small by physical boundaries that do not permit mass exchange with a bulk reservoir of A, except for a small region, for example, with a size on the order of that of A. In this scenario, an ensemble of configurations with inconsistent set of parameters specifying the system emerges because the constant chemical potential, or constant concentration, of A is only partially observed. Albeit not conforming to any thermodynamic ensemble, one can argue that such a system can be represented as a hybrid between canonical and grand-canonical ensembles. Although this may be possible to approximate by interpolation, we do not attempt to address this case here and limit our derivation only to the canonical ensemble which is completely closed to mass transfer.

#### 2.4.2 Relation between the Reference and Arbitrary Systems

In deriving the equilibrium constant of adsorption, we required the reference system to be large (macroscopic). This is necessary in order to apply a thermodynamic relation (eq 6), as well as Stirling’s approximation (eq 7), for the reference system, so that the corresponding Gibbs free energy change, per mole (Δ*G*^⊖^), can be expressed in terms of single-particle and pair-particle partition functions. In contrast, no assumption on the size of the arbitrary system was made, and hence, its size can be either large or small. It is only required that the arbitrary and reference systems are at the same temperature, but aside from that, the former can accept any set of *N*_A_^total^, *N*_S_^total^, and *V* values, including the smallest system possible that consists of one adsorbate molecule and one adsorbing site. These arbitrary and reference systems are linked together by eq 9, enabling the Gibbs energy change of full conversion of the reference reaction to be expressed by concentrations of reactants and product, observed at equilibrium, of any arbitrary system, that is, by the equilibrium constant given in eq 14. It is this property of the equilibrium constant, which reports on the Gibbs energy change of the reference reaction and not on the Gibbs energy change of the arbitrary reaction (i.e., at the arbitrary conditions), that makes *K* so important in chemical equilibrium. Because then, its value is constant by definition (eq 3) and at the same time, it can be calculated from any arbitrary system. Expressed the other way around, equilibrium composition of a chemical reaction at any arbitrary conditions (except the temperature) can be predicted if we know the value of *K*.

#### 2.4.3 Experimental Realizations of Closed Small Systems

Testing the predictions made in this paper requires the ability to monitor localizations of mass at the single-molecule level. Technically, such capability was reported three and a half decades ago in crystals^67^ and soon after in solutions.^68^ An additional requirement is the capacity to confine the monitored molecules to a small system, normally characterized by a small volume. This can be realized by several methods. For example, surfactant-stabilized aqueous droplets can form confined “containers” with pico-to atto-liter volume^69−74^ wherein reactions involving small numbers of chemical components can be followed, usually with fluorescence microscopy.^75,76^ Another example is imaging the behavior of biomolecules in living cells^77^ and exosomes.^78^ In this respect, the use of synthetic vesicles such as liposomes, which are widely utilized as pharmaceutical nanocarriers,^79^ can provide better control on the identity and concentrations of the different encapsulated molecules.^80−82^ To increase accuracy in reading fluorescence signals, the liposomes in bulk solution are often immobilized by surface tethering.^83^ Of a particular interest to the proposed statistical analysis is the embedding of transmembrane proteins across the lipid bilayer membrane of the vesicle. On that note, the surface density of the proteins can be controlled by adjusting the protein/lipid ratio when preparing the vesicles.^84−87^ In these systems, the proteins’ cytosolic receptors can bind with encapsulated ligands at varying concentrations. Being immobile within the lipid bilayer structure, these receptors can be identified as surface sites and the ligands as adsorbate molecules in the adsorption model proposed in this paper. Attention should be given that the ligand is at low concentration and the membrane protein is at low surface density, so that their ideal behavior is not compromised.

#### 2.4.4 Application to an Experimental System

We now elaborate on a specific closed small system in which *N*_A_^total^ = *N*_S_^total^ = 1. Here, there are only two possible macroscopic states in the system, one corresponding to the adsorbed state, SA, and the other to the unadsorbed state, A + S. If the fluorescence emission signals indicate the fraction of time, thus the probability, of observing the adsorbed state is *p*^SA^ = ⟨*N*_SA_⟩ (which means that the fraction, or probability, of the unadsorbed state is *p*^A+S^ = 1 – *p*^SA^ = ⟨*N*_A_*N*_S_⟩), the expression of *K* in eq 14 becomes^42,57^
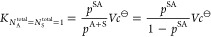
23This system, in which one adsorbate molecule interacts with one binding site, has been constructed experimentally for tracking the kinetic and thermodynamic behavior of single molecules. One of the advantages of such a system is that fluorescence resonance energy transfer (FRET) measurements are facilitated.^88^ More specifically, in order to increase reading accuracy in FRET experiments, the fluorescence signals ought to be spatially separated, limiting the studied systems to those containing low concentrations of interacting particles, which in turn restrict the investigations to adsorbate–adsorbent (or protein-receptor) pairs with large binding affinities. However, if these chemical species are encapsulated inside a vesicle with a small volume (e.g., a diameter of 100 nm yields approximately an atto-liter volume), their concentrations can be large, but at the same time, the optical signals can be spatially well resolved provided the distance between the surface tethered vesicles are large enough. For example, Chen and co-workers^89,90^ studied the interactions between the copper chaperone Hah1 protein and Wilson disease protein. The latter is a multidomain protein that is anchored to organelle membranes. The preparation of the nanovesicles was designed to encapsulate only one pair of proteins, and the analyses of the data were performed only from vesicles adhering to this content. In addition, nonspecific interactions between the encapsulated proteins and the lipid membrane were found to be insignificant, indicating an ideal behavior of the nanosized system. To obtain the bimolecular dissociation constant between A and B proteins, the following expression, *K*_D_ = (*p*^A+B^/*p*^AB^)(1/*V*), was used. Apart from the standard concentration (introduced to render the equilibrium constant unitless), this expression is the reciprocal of the binding constant described in eq 23. Likewise, in calculating the bimolecular reaction rate constant,^89,91,92^*k*, the observed reaction rate, d(*c*_AB_)/d*t*, is equated to the term *k*⟨*c*_A_⟩(1/*V*). We emphasize that the expressions utilized for the dissociation constant and bimolecular rate constant are not the same as those known from chemistry textbooks. The authors of the experimental studies argue that “for the single-molecule reaction occurring in a nanovesicle”, the concentration of one of the particles (B) should be substituted by the term 1/*V* which represents an “effective concentration of one molecule inside the nanovesicle”. Our interpretation is that this term follows from the requirement to take into account cross-correlations in concentrations, that is, *K*_D_ = ⟨*c*_A_*c*_B_⟩/⟨*c*_AB_⟩, and the rate of product formation equals *k*⟨*c*_A_*c*_B_⟩. Then, for a system with *N*_A_^total^ = *N*_S_^total^ = 1, the two-body average, ⟨*c*_A_*c*_B_⟩, reduces to a one-body average, ⟨*c*_A_⟩(1/*V*). Note also that in this case, the probabilities are proportional to the corresponding concentrations, *p*^AB^ = *V*⟨*c*_AB_⟩ and *p*^A+B^ = *V*⟨*c*_A_⟩ = *V*⟨*c*_B_⟩ = *V*^2^⟨*c*_A_*c*_B_⟩.

## 3 Conclusions

Due to their large amplitudes of fluctuations, properties of finite systems can be determined only by averaging over time or over configurations, and because adsorption is a two-body process, averaging its reaction rate necessitates the inclusion of cross correlations in reactant’s concentrations. For this reason, Langmuir’s equation breaks down when the numbers of adsorbate molecules and/or adsorbing sites are small. In this paper, we derived a general expression of the equilibrium constant for adsorption, *K*, that is valid also at small scales for closed systems. Despite the distinguishable character of the adsorbing sites, the expression obtained is the same as that for binding reactions where both reactants are indistinguishable particles. Moreover, it is shown that this expression of *K* yields values that are constant upon changes in concentrations and system’s size, down to the smallest system possible. In addition, we present an alternative equation to the Langmuir adsorption isotherm where the expression of the fluctuations, *l*(*N*_SA_, *N*_SA_), is approximated by interpolation between two extreme cases that can be solved exactly; the thermodynamic limit and small systems where the particle number of at least one reactant equals one. Given the value of the equilibrium constant and total number of adsorbing sites, *N*_S_^total^, this proposed adsorption equation (eqs 17, 19, and 21) predicted almost perfectly the fraction of occupied sites observed by four series of simulations modeled by the MC technique. Note that in contrast to Langmuir’s equation (eq 2), eq 17 also requires knowledge of the system’s volume, *V*. Nonetheless, when *V*, *K*, and *N*_S_^total^ are not available, the amount of molecules adsorbed, ⟨*N*_SA_⟩, can be plotted as a function of the total amount of adsorbate molecules introduced into the system, *N*_A_^total^. Then, the (nonlinear) curve fitting can consider the term *Vc*^⊖^/*K* as a single parameter which, together with *N*_S_^total^, reduces the number of fitted parameters to two.

## 4 Materials and Methods

**Figure 6:**
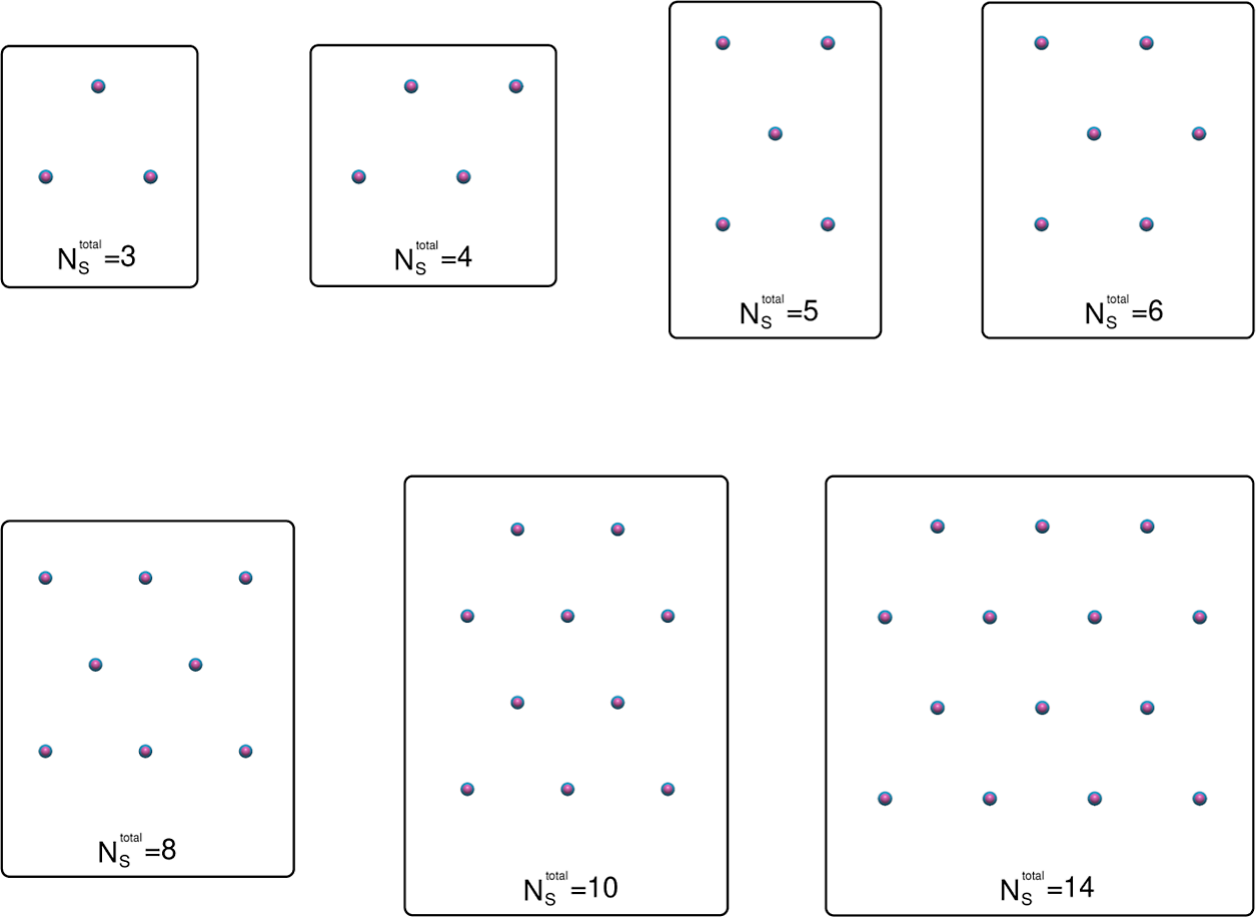
Configurations of immobile adsorbing sites *S* for 3 ≤ *N*_S_^total^ ≤ 14 projected onto the *xy*-plane. *s* particles are depicted in magenta whereas *h* particles are depicted in blue. All sites have the same *z*-coordinates forming a two-dimensional equilateral triangular lattice with the nearest neighbor distance of 3.5 nm. The configurations for *N*_S_^total^ = 1,2 are trivial, those for *N*_S_^total^ = 30 (120) are built by 6 (12) rows of 5 (10) sites, whereas that for *N*_S_^total^ = 68 is built by 5 rows of 8 alternating with 4 rows of 7 sites.

The model system consists of *N*_S_^total^ adsorbing sites, S, each composed of two particles, *s* and *h*, whose Cartesian coordinates were fixed throughout the simulations. *x*- and *y*-coordinates of *s* and *h* particles were the same and correspond to a two-dimensional equilateral triangular lattice. *z*-components of all *s* particles equaled 2.50 *nm*, coinciding with the midpoint (along the *z*-axis) of the rectangular simulation box. The *h* particles were placed at *z* = 2.64 nm, thus 0.14 nm away from the s particles, and functioned as protecting groups to prevent binding of more than one adsorbate to a single adsorbing site. Nearest neighbor distances between S sites equaled 3.5 nm and the shape of the triangular lattice formed by the *N*_S_^total^ sites was chosen to generate, as much as possible, equal dimensions along the *x*- and *y*-axes (see Figure 6).

*N*_A_^total^ adsorbate molecules, A, in the gas phase are introduced randomly into the simulation box. Each A molecule is composed of two atoms, *a* and *h*, “covalently” bonded with a bond length of 0.14 nm. All atom sites in the system have zero charge, *q*_s_ = *q*_a_ = *q*_h_ = 0.0 *e*, and their intermolecular interactions are described by LJ potentials truncated at a distance of 2.0 *nm*. LJ parameters, σ and ϵ, for the interactions between different atom sites are specified in Table 1. With these parameters, all interactions are effectively repulsive except for a strong attraction between the *s* and *a* atoms, resulting in adsorptions of one A molecule onto one S surface site, and this adsorption site (whether occupied or nonoccupied) does not interact with any other surface sites. To define a state of an occupied (bound) site, a cutoff value of the interparticle distance between *s* and *a* is utilized, *r*_sa_ < 0.37 nm, which captures the width of the first maximum (observed at *r*_sa_ = 0.204 nm) in plots of all *g*_sa_(*r*)’s. With this cutoff distance, the number of times in which two A molecules were counted as occupying the same S site was negligible. More explicitly, these “doubly occupied-sites” incidents were recorded only in the R1 series for *N*_S_^total^ = *N*_A_^total^ ≥ 30 with average numbers smaller than 3 × 10^–6^.

**Table 1:** Intermolecular LJ Parameters between Immobile Adsorbing Sites, *S*(*sh*), and Adsorbate Molecules, *A*(*ah*), and between the Adsorbate Molecules Themselves

	σ [nm]	ϵ [kJ/mol]
*a*···*a*	0.75	0.1
*h*···*h*	0.50	0.1
*s*···*h*	0.35	0.1
*a*···*h*	0.35	0.1
*s*···*a*	0.18	35.0

All simulations were performed in the canonical (*N*_A_^total^, *N*_S_^total^, *V*, *T*) ensemble with *T* = 300 *K*. Total numbers of A particles and S sites, as well as volume, were varied systematically in different simulations. Changes in volumes were achieved by modifying the length of the rectangular simulation box along *x*- and *y*-axes, *L*_*x*,box_ = *L*_*y*,box_, while maintaining *L*_*z*,box_ = 5.00 nm constant. Four series of simulations were constructed. In the first, R1, the value of *N*_A_^total^ = *N*_S_^total^ equaled 1, 2, 3, 4, 5, 6, 8, 10, 14, 30, 68, and 120 keeping the concentration *c*_A_^total^ = *N*_A_^total^/*V* constant at 0.008 molecules/nm^3^ (∼0.013 M). Thus, values of *L*_*x*,box_ = *L*_*y*,box_ ranged from 5.0 nm for the smallest system to 54.77 nm for the largest system. In the second series of simulations, R2, the number of surface sites was fixed, *N*_S_^total^ = 4, whereas *N*_A_^total^ varied from 1 to 120. On the other hand, in the third series, R3, *N*_A_^total^ = 4 is fixed while *N*_S_^total^ ranged from 1 to 120. In both R2 and R3, the concentration of the most abundant species, *c*_S_^total^ or *c*_A_^total^, is kept constant at 0.015 molecules/nm^3^ (∼0.025 M). We also performed simulations, R4 series, in which the adsorption energy is systematically varied. To this end, the LJ parameter ϵ between *s* and *a* atom sites (ϵ_SA_) increased from 15.0 to 50.0 kJ/mol in locksteps of 5.0 kJ/mol, keeping all other parameters in the system the same as indicated in Table 1. We chose to conduct R4 series with *N*_S_^total^ = *N*_A_^total^ = 2 at *c*_A_^total^ = 0.008 molecules/nm^3^ (thus, *L*_*x*,box_ = *L*_*y*,box_ ≃ 7.07 nm) because this system exhibits the largest deviation with our previously proposed prediction of surface coverage.

Generations of different system’s configurations forming a canonical ensemble were done by the MC method,^93,94^ coded in-house and ran in double-precision arithmetic. Periodic boundary conditions were applied along all three Cartesian axes. The Metropolis acceptance criterion^95^ was applied to either accept or reject trial moves. Each trial move is composed of randomly selecting one A molecule which is then displaced, in each of the three Cartesian axes, and rotated around each of the two axes perpendicular to the molecular axis. These displacements and rotations are performed as rigid bodies. Their magnitudes and directions were determined randomly from a uniform distribution with maximum values of 0.4 nm for displacements along each of the Cartesian axes, 0.1 for cos θ when rotating around angle θ (0 ≤ θ ≤ π), and 0.314 rad for rotations around angle ϕ (0 ≤ ϕ ≤ 2π). These trial moves resulted in acceptance ratios that for R1–R3 series varied from 0.162 (R2, *N*_A_^total^ = 1) to 0.964 (R2, *N*_A_^total^ = 120), and for R4 series ranged from 0.006 (ϵ_SA_ = 50.0 kJ/mol) to 0.993 (ϵ_SA_ = 15.0 kJ/mol). For all systems, at least 5 × 10^9^ trial moves were taken for equilibration. The number of trial moves for data collection was, approximately, inversely proportional to the size of the system; more specifically in the R1 series, data was collected by 1.0 × 10^12^ trial moves for the smallest system and by 1.2 × 10^10^ trial moves for the largest system. In R2 and R3 series, these numbers ranged from 5.0 × 10^11^ to 2.4 × 10^10^ trial moves, whereas in R4 series, it equaled 1.2 × 10^11^ for all simulations.

## Supporting Information Available

Application of currently proposed approximation to MC simulation results performed in a previous study (PDF)
