# Supplementary material for: Breakdown of Langmuir Adsorption Isotherm in Small Closed Systems
Source: Langmuir. 2024 Feb 5;40(7):3900–10. doi: 10.1021/acs.langmuir.3c03894 (PMC10883037; doi:10.1021/acs.langmuir.3c03894)
Supplement: Supplementary file 1 — la3c03894_si_001.pdf [file la3c03894_si_001.pdf]

Supporting Information: Breakdown of Langmuir Adsorption Isotherm in Small Closed Systems

Ronen Zangi*[1,2,3]

[1]*Donostia International Physics Center (DIPC), 20018 Donostia-San Sebastián, Spain*

[2]*Department of Organic Chemistry I, University of the Basque Country UPV/EHU, 20018 Donostia-San Sebastián, Spain*

[3]*IKERBASQUE, Basque Foundation for Science, 48009 Bilbao, Spain*

January 9, 2024

*r.zangi@ikerbasque.org

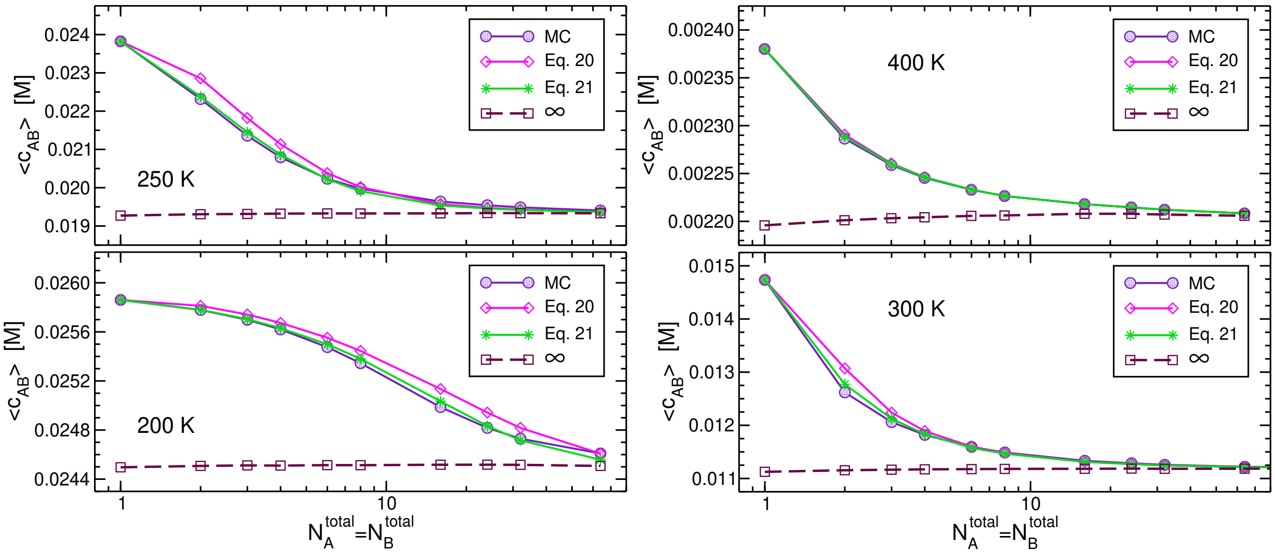

Figure SI-1: Application of currently proposed approximation to results obtained in a previous study of binding reactions, $A + B \rightleftharpoons AB$ (see Fig. SI-3.5 in Supplementary information[1]). Average concentrations of bound particles, $\langle c_{AB} \rangle$, are calculated by K using Eq. 17 wherein the relative fluctuations $l(N_{AB}, N_{AB})$ are approximated by Eq. 19 with λ given by Eq. 21 (green, stars). Evaluation of λ by Eq. 20 corresponds to a previously proposed approximation (magenta, diamonds). Concentrations calculated directly from the MC simulations are shown as references (violet, circles). The dashed maroon lines (squares symbols) are the corresponding values at the thermodynamic limit, $l(N_{AB}, N_{AB}) \to 0$, calculated at each value of $N_A^{\text{total}} = N_B^{\text{total}}$. For temperatures in the range $500 - 1200\ K$, both predictions are more accurate than those exhibited at $T = 400\ K$ (graphs not shown). At $T = 300\ K$, the actual curves end at $N_A^{\text{total}} = N_B^{\text{total}} = 4096$, however, the last four points are not shown because the predictions obtained are more accurate than that of the last point displayed at $N_A^{\text{total}} = N_B^{\text{total}} = 64$.

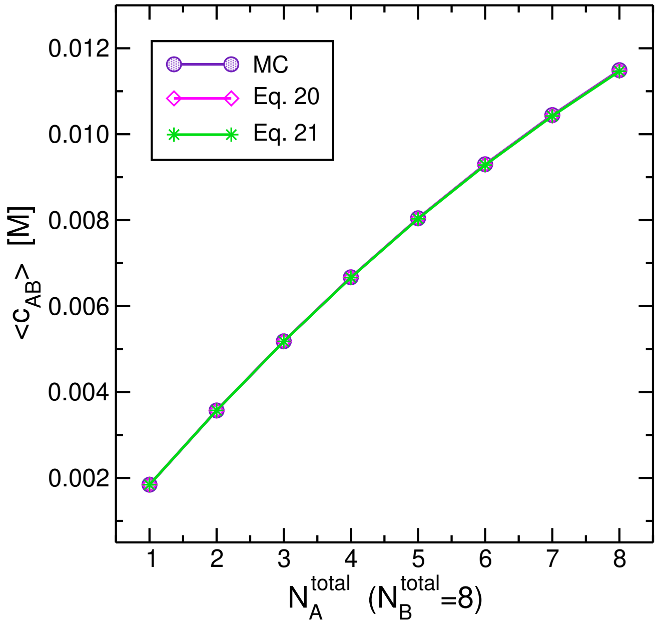

Figure SI-2: Another application of the currently proposed approximation to results obtained in a previous study of binding reactions, $A + B \rightleftharpoons AB$ (see Fig. SI-3.6 in Supplementary information[1]). In these simulations, N_A^{total} and N_B^{total} are not equal and N_A^{total} is not fixed at the value of 1. More specifically, N_A^{total} varied from 1 to 8, whereas $N_B^{\text{total}} = 8$, $V = 512\ nm^3$, and $T = 300\ K$ are fixed. Curves' colors and symbols are the same as those in Fig SI-1.

References

[1] Zangi, R. Binding Reactions at Finite Systems, *Phys. Chem. Chem. Phys.* **2022**, *24*, 9921–9929.
